# National Medical Convention

**Published:** 1846-06

**Authors:** 


### THE WESTERN JOURNAL.

NEW SERIES.—VOL. V.—NO. VI.

LOUISVILLE, JUNE 1, 1846.

### NATIONAL MEDICAL CONVENTION.

This body assembled, Tuesday the 5th of May, at the University Medical College, in the city of New York. The assemblage embraced one hundred and twenty seven members, from sixteen States. The medical schools represented in the Convention were, the two schools of the city of New York, the Geneva Medical College, the Vermont Medical College, the Berkshire Medical Institute, in Massachusetts, Medical Institute of Yale College, the Albany Medical College, the Pennsylvania Medical College, the Washington Medical College of Baltimore, La Porte University, the Illinois Medical College, the University of St. Louis, and the Franklin Medical College. Among the teachers in medical schools, we remark the names of Professors Pattison, Bedford, Parker, Knight, Lee, Beck, Patterson, Atlee, Baxley, Pope, and Clymer. Several editors took part in the deliberations of the Convention, among whom were Dr. John Bell, Dr. Hays, Dr. Fenner, Dr. Lee, and Dr. Flint.

A temporary organization, preparatory to the regular proceedings of the Convention, was effected by the nomination of Dr. Bell, of Philadelphia, as Chairman, and Dr. Buell, of New York, as Secretary. Dr. Knight, of New Haven, was elected President of the Convention; Dr. Bell, and Dr. Delafield, Vice Presidents; and Dr. Stillé, of Philadelphia, and Dr. Arnold, of Savanah, Secretaries.

Dr. Bedford offered the following resolution which he accompanied by some remarks intended to show that it was useless to proceed unless the convention were what in truth it ought to be, and what it is called, a *National Convention.*

"Whereas, the object of this Convention has not been carried out; and whereas, a full representation from the Medical Colleges and Universities is necessary; and whereas, the call has been defeated by the absence of the necessary members from the various parts of the Union, that, therefore, this Convention do adjourn *sine die.*"

This motion was lost by a vote of 74 to 2; whereupon Dr. Clymer, of Philadelphia, offered the following resolution:

"*Resolved,* That a Committee be appointed to provide other accommodations than the present for the sittings of this Convention."

Dr. Beadell, of New York, moved as an amendment, "that this Convention do immediately adjourn to the rooms of the College of Surgeons and Physicians." Dr. Bedford made a brief explanation, in which he disavowed any sentiments of hostility or disrespect towards the Convention. Dr. Pattison followed his colleague in the same strain, and concluded by remarking, that, after these explanations, "if the Convention think right to refuse the courtesies and civilities of the Faculty of the University of the City of New York, all he had to say was, 'God speed them.'" The disclaimers were esteemed satisfactory, and the removal did not take place.

The Convention was in session two days, and when it adjourned it did so to meet at Philadelphia in May next. Among the resolutions adopted we note the following, as among the most important. They were offered by Dr. Hays, editor of the American Journal of the Medical Sciences, and seconded by Professor Pattison.

"*Whereas,* It has been shown, by experience, that the association of persons engaged in the same pursuit, facilitates the attainment of their common objects; therefore,

"*Resolved,* That it is expedient for the medical profession of the United States to institute a National Medical Association, for the protection of their interests, for the maintenance of their honor and respectability, for the advancement of their knowledge, and the extension of their uselfulness.

"*Resolved,* That a committee of seven be appointed to report a plan of organization for such an association, at the meeting to be held in Philadelphia on the first Wednesday in May, 1847.

"*Resolved,* That a committee of seven be appointed to prepare and issue an address to the different regularly organized Societies and chartered Medical Schools in the United States, setting forth the objects of the National Medical Association, and inviting them to send delegates to a Convention to be held in Philadelphia, on the first Wednesday in May, 18[illegible]7.

"*Resolved,* That it is desirable that a uniform and elevated standard of requirements for the degree of M. D., should be adopted by all the medical schools in the United States, and that a committee of seven be appointed to report on this subject at the meeting to be held in Philadelphia on the first Wednesday in May, 1847.

"*Resolved,* That it is desirable that young men, before being received as students of medicine, should have acquired a suitable preliminary education, and that a committee of seven be appointed to report on the standard of the acquirement which should be exacted of such young men, and to report at the meeting to be held on the first Wednesday in May, 1847.

"*Resolved,* That it is expedient that the medical profession in the United States should be governed by the same code of medical ethics, and that a committee of seven be appointed to report a code for that purpose at the meeting to be held at Philadelphia on the first Wednesday in May, 1847."

We are truly gratified at the results of the Convention, and especially at the perseverance manifested by its members. They were not discouraged by the partial attendance, but resolved to hold on and hold out, until more interest should be awakened in the minds of the profession. We predict that the next Convention will be more generally attended,—that the medical schools and societies will be more numerously represented, and that great benefits will yet flow from these assemblages.

We have compiled this hasty sketch of the proceedings of the Convention from the New York papers. A more detailed report may soon be expected, the Convention having decided to publish its proceedings in pamphlet form, and to distribute ten thousand copies of the same among the profession.

